# Impact of COVID-19 on Ethnically Minoritised Carers in UK’s Care Home Settings: a Systematic Scoping Review

**DOI:** 10.1007/s40615-023-01640-3

**Published:** 2023-07-06

**Authors:** Paul Wesley Thompson

**Affiliations:** Kellogg College, University of Oxford, Oxford, UK

**Keywords:** COVID-19, Care home workers, PPEs, Mental health, Ethnically minoritised

## Abstract

COVID-19 has impacted disproportionately two groups in the UK: healthcare workers and people from ethnically minoritised groups. However, there is a lack of evidence on how COVID-19 affected ethnically minoritised carers in care homes. Therefore, the present study aimed to explore the available evidence regarding the impact of COVID-19 on ethnically minoritised carers in UK. The relevant records were systematically searched in Cochrane COVID‐19 Study Register and WHO COVID‐19 global literature. A total of 3164 records were retrieved. Following duplicate elimination and abstract, title, and full-text screening, 10 studies were identified as eligible for the present scoping review. Most of the studies were conducted in the UK and USA, involving diverse healthcare occupations and methodologies. Multiple studies found anxiety, depression, stress, and post-traumatic stress disorder among carers with high odds among ethnically minoritised carers. Limited access to personal protective equipment and workplace discrimination was noted and linked with poor mental health. The carers reported difficulties in care delivery and managing extra workload arising from staff shortages. The risk of infection and clinically significant mental disorders was higher among carers from the ethnically minoritised background. They exhibited fear about care homes’ uncertain futures and consequential financial losses. Conclusively, COVID-19 appeared to exert adverse effects on practices and experiences of ethnically minoritised carers in the UK’s care homes; however, further studies are warranted to increase the understanding of COVID-19-related experiences of this group of carers which significantly contribute to the country’s healthcare system.

## Introduction

Coronavirus disease 2019 (COVID‐19) is a novel disease caused by a strain of coronavirus known as severe acute respiratory syndrome coronavirus 2 (SARS‐CoV‐2). Its outbreak was declared a pandemic in March 2020 by World Health Organization [1]. The virus has since spread rapidly, causing millions of infections and deaths across the globe [2]. The manifestation of COVID-19 varies from asymptomatic patients to organ dysfunction [3]. However, COVID-19-related incidence, severity, and mortality discrepancies have been reported across various subpopulations [4]. Research has reported higher proportions of COVID-19-positive individuals among minorities compared to their White counterparts. Black individuals exhibited higher 30-day in-hospital mortality than non-Black persons [5, 6]. The likeliness of COVID-19-related deaths in Black males and females of the UK was respectively recorded at 4.2 times and 4.3 times higher than males and females of White ethnicity [7].

Besides higher incidence and severity in ethnically diverse minorities, COVID-19 appears to impact more intensely on workers from occupations that involve close proximity during daily functions. For instance, health, education, food, retail, transportation, and others [8, 9]. Among these, healthcare workers remained at the heart of the pandemic owing to their role in the frontline diagnosing and treating growing numbers of acute and critically ill COVID-19 patients [10]. Data analysis involving 902,813 workers of National Health Service (NHS) trusts reported up to 2.5 times the relative risk of COVID-19 for individuals in patient-facing occupations [11]. Moreover, the healthcare segment faced challenges in managing COVID-19 due to a lack of preparation in dealing with the pandemic, limited awareness of new emerging COVID-19 strains, and a shortage of staff arising from COVID-19-related restrictions, precautions, and workload. These led to mental and physical exhaustion among healthcare workers [12].

Care home workers (CHWs) have increased exposure due to their lead involvement with residents’ care [13]. In the UK, patients were discharged to care homes from NHS hospitals after treatment for COVID-19 infection, where they could be provided for their wide-ranging complex needs. Inadequate testing facilities and availability of personal protective equipment (PPE) further complicated the working situations in UK’s care homes at the onset of COVID-19 [14]. The risk factor was further substantiated by many Black carers employed at care homes [15]. The care home sector in the UK is made up of a significant number of ethnic minorities who have been exposed to substantial risk in their day-to-day work, particularly during the early days of the pandemic. Ethnic minorities make 23% of England’s adult social care workforce; about half of this workforce is accounted for by carers from Black/African/Caribbean/Black British backgrounds [15]. Black and other ethnically minoritised workers have slightly high probability of being disproportionately represented in the health and social care sectors of the UK [16]. According to media reports during early phase of COVID-19 in UK, 72% of all health and social care staff who died from COVID-19 were ethnically minoritised [17]. Various studies have been conducted to understand the impact of COVID-19 on healthcare professionals in hospital settings [18–22]; however, the research is limited [23] regarding the exploration of carers’ experiences in care homes during COVID-19. Moreover, the impact of COVID-19 on ethnically minoritised carers has not been reported in detail.

Therefore, the present study aimed to explore the existing knowledge on the experiences of ethnically diverse carers during COVID-19. A scoping review was conducted to chart the available evidence and identify the research gaps in knowledge regarding the impact of COVID-19 on ethnically minoritised carers in UK’s care homes.

## Method

A scoping review was conducted as per the guidelines of the Joanna Briggs Institute Manual for Evidence Synthesis [24] and Preferred Reporting Items for Systematic Reviews and Meta-Analyses Extension for Scoping Reviews (PRISMA-ScR) checklist [25] for reporting.

### Research Question

The current scoping review aimed to address the following key question: what evidence is available on the UK’s ethnically minoritised carers’ experiences of COVID-19?

### Search Strategy and Study Selection

Using the SPIDER tool (Table 1), the keywords *care* OR *home* OR *homes* OR *worker* OR *workers* were separately combined with *ethnicity*, *ethnicities*, *ethnic*, *minority*, *minorities*, *Black*, *Blacks*, *Asian*, *Asians*, and *ethnically minoritised* using AND as Boolean in Cochrane COVID‐19 Study Register (CCSR) and WHO COVID‐19 global literature. Because both databases are dedicated to indexing COVID-19-related human and primary studies, the term ‘COVID-19’ and related synonyms were not combined with search keywords. The results were retrieved on 22^nd^ May 2022 as separate files for each search string without using any filters and were imported to the EndNote X8 using its built-in duplicate removing function. The relevancy of studies was determined by reading their title and abstracts. The full texts of selected studies were obtained and reviewed for their eligibility according to the SPIDER tool, inclusion criteria, and exclusion criteria.

**Table 1:** SPIDER tool for search terms

S	Sample	Carers from ethnically minoritised background
PI	Phenomenon of interest	Impact/experience of the sample regarding COVID-19
D	Design	Any qualitative/quantitative/mixed methods design
E	Evaluation	Impact, experiences, perceptions, satisfactions, observations
R	Research type	Qualitative/quantitative/mixed methods

#### Inclusion Criteria

The studies published in the English language with qualitative, quantitative, or mixed methods design reporting the impact, experiences, perceptions, satisfactions, or observations and sample size of ethnically minoritised carers of UK’s care homes during COVID-19 were included in the present scoping review.

#### Exclusion Criteria

The following exclusion criteria were applied during the identification of the relevant studies: the studies published in a language other than English, from non-UK-based care home settings, involving participants from care home settings situated in a country other than the UK, and not providing the distribution of ethnically minoritised carers.

### Data Extraction

The data from identified studies were extracted into a table which included the following headings: authors, year of publication, study design, sample size of all and carers from the ethnically minoritised background, occupation type, and brief results.

## Results

### Results of the Search

**Fig. 1:**
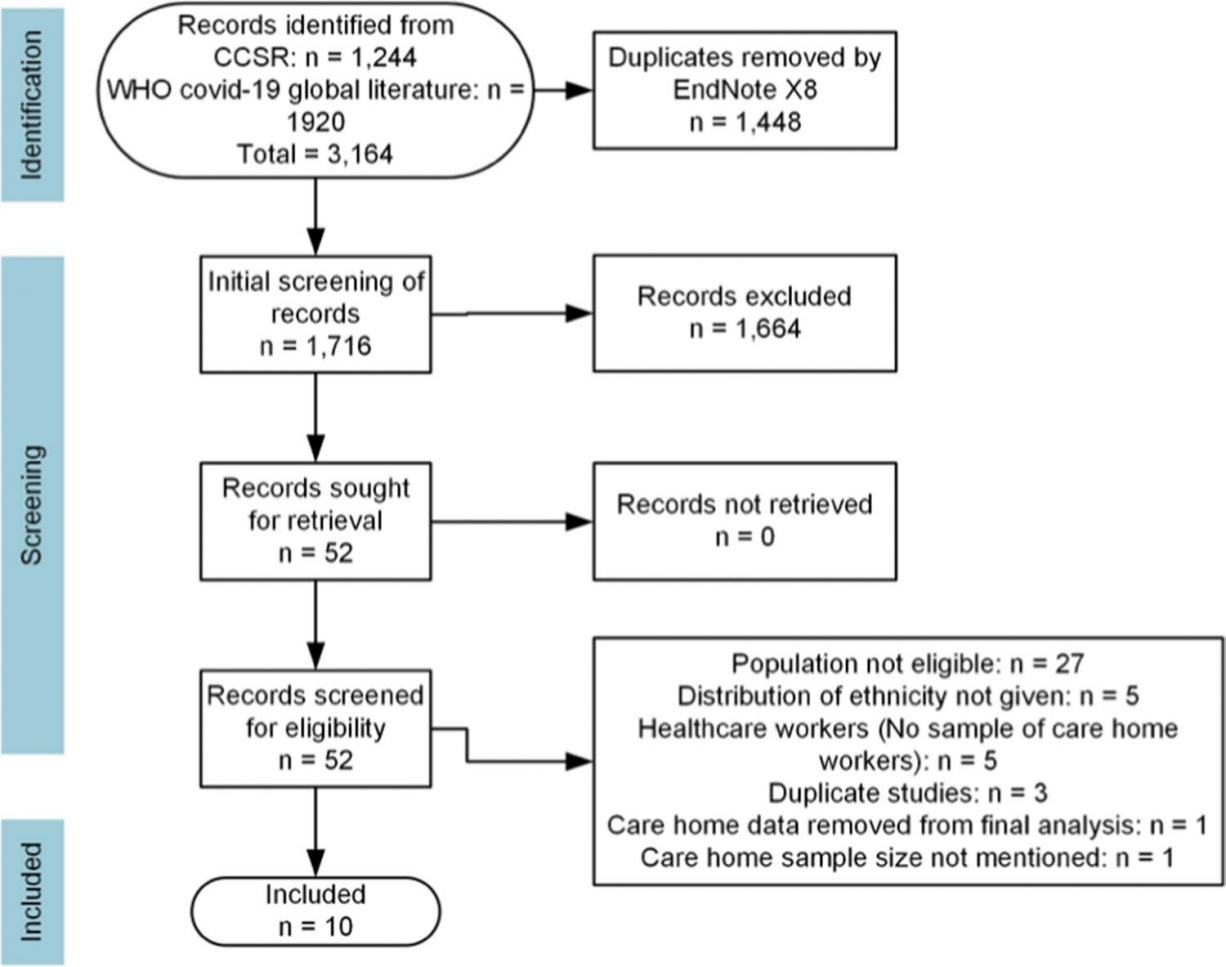
PRISMA flowchart for identification of studies via databases

The search in CCSR resulted in 1244 records, while 1920 were retrieved from the WHO COVID-19 global literature. During import to EndNote, 1448 duplicate references were removed from total 3164 records. The title and abstract screening resulted in the selection of 52 studies. Following their full-text screening, 10 eligible studies were included in the present scoping review. The process and results of study selection are presented in the PRISMA flow diagram (Fig. 1).

### Characteristics of Studies

**Fig. 2:**
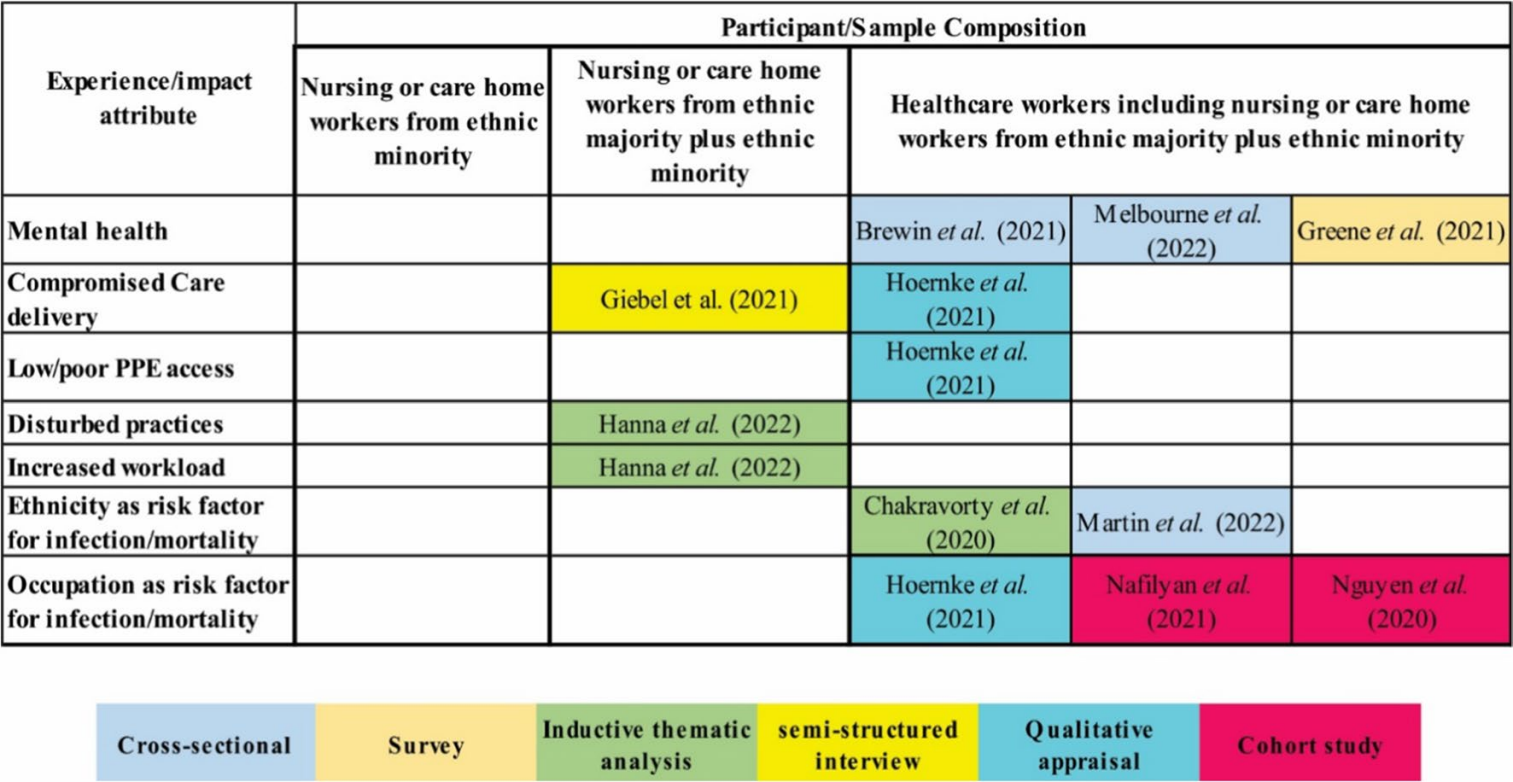
Map of the studies according to outcome categories, study types, and population categories showing evidence gap

The data from included studies under various headings are summarised in Table 2. The map of the distribution of studies related to the outcome categories, study types, and population categories is illustrated in Fig. 2.

**Table 2:** Summary of included studies

Author (s) Year of publication	Study design	Sample size (total) and ethnic minorities	Occupation type (sample size)	Main outcome measures	Brief result/conclusion
Brewin et al. [26] 2021	Cross-sectional national online survey	1033 ethnically minoritised (84)	Nursing or care home (157)	Tipping point for severe anxiety or depression	Approximately 50% of the respondents who opted ‘yes’ for at least one question met the threshold, while the power of positive prediction raised from 0.49 to 0.72 for the responders who responded ‘yes’ to both questions
Melbourne et al. [27] 2022	Cross-sectional analysis	11,695 Black (491) Asian (2223) Mixed/Multiracial (495) Other (245)	Nursing or care home (273)	Anxiety or depression symptoms and PTSD	The odds of PTSD were higher in Black, Asian, mixed/multiple, and other ethnic groups as compared to White ethnic group Ethnic minority experienced PTSD-, anxiety-, and depression-associated work and sociodemographic factors
Greene et al. [28] 2021	Online questionnaire-based survey	1194 Black (18) Asian (39) Multiracial (25) Other (18)	Nursing or care home (177)	PTSD, depression, and anxiety	The respondents working in nursing or care home settings exhibited significantly higher levels of PTSD symptoms in comparison to other work settings
Hanna et al. [29] 2022	Semi-structured interview-based qualitative study	16 ethnically minoritised (1)	Care home (16)	Impact of COVID-19 on the working practices of care home staff	COVID-19 significantly disturbed routine practices and forced the staff to adopt extra duties on top of increased workloads
Giebel et al. [23] 2021	Semi-structured interview-based qualitative study	16 (1)	Care home (16)	Impact of COVID-19 on care delivery and visits from family members and staff	The staff struggled to balance between ensuring mental well-being of residents and effectively controlling infection spread
Hoernke et al. [30] 2021	Cross-sectional qualitative analysis	-	-	Experiences with PPE	All streams of data analysis found reports of PPE shortages throughout UK, particularly in care homes, community health facilities, and general practice
Chakravorty et al. [31] 2020	Survey	2003 ethnically minoritised (1732)	Care homes (63)	Exploration of COVID-19-realted emerging concerns	Ethnically minoritised background appeared as independent risk factor for COVID-19 infection
Nafilyan et al. [32] 2021	Cohort study	14,295,900 Black (414,440) Asian (704,451) Mixed (172,402) Other (363,548)	Care workers and home (397, 620)	Occupation and mortality	Care workers and home exhibited third highest age-standardised mortality rates
Martin et al. [33] 2022	Cross-sectional analysis	12,541 Black (535) Asian (2418) Mixed (529) Other (264)	Nursing or care home (242)	COVID-19 risk factors in a multiethnic cohort of UK	Risk of infection was higher among Black than that in White
Nguyen et al. [34] 2020	Cohort study through COVID Symptom Study smartphone application in UK and USA	Frontline (99,795; 85.4% from UK and 14.6% from USA) Black (1197) Asian (4390) Multiracial or other (2395)	Not mentioned	COVID-19 risk among frontline	The risk was greatest in nursing homes (where inadequate PPE was reported) and in inpatient settings (where providers PPE reuse was reported)

### Study Types and Settings

Among included studies, three were designed as cross-sectional studies [26, 27, 33], two cohort studies [32, 34], two qualitative studies [23, 29], and two studies were online surveys [28, 31]. One cross-sectional study conducted a qualitative analysis of data from different sources regarding experiences of PPE [30]. The majority of included studies collected data through online interviews or surveys [23, 26–31]. Martin et al. [33] and Nafilyan et al. [32] performed cross-sectional analysis and cohort using data from other larger studies. Many studies recruited participants from different healthcare occupations; only two thematic analyses of semi-structured interview-based studies enrolled carers from care homes only [23, 29].

### Population

The included studies mainly involved healthcare, allied healthcare, and health support workers from hospitals, representing a comparatively lower population from care or nursing homes. Only two studies were conducted specifically on participants from care homes [23, 29]. The studies collectively characterised the participants’ ethnic distribution as ethnically minoritised [23, 26, 29, 31] or separately into Black, Asian, Mixed/multiracial, and ‘other’ groups [27, 28, 32–34].

### Outcomes and Outcome Measures

Greene et al. [28] utilised Generalised Anxiety Disorder Scale-7 and International Trauma Questionnaire to assess anxiety and post-traumatic stress disorder (PTSD), respectively. Brewin et al. [26] evaluated depression and anxiety among carers through Patient Health Questionnaire-9 and Generalised Anxiety Disorder Scale-7. Melbourne et al. [27] employed Patient Health Questionnaire-2 scale for depression, PTSD Checklist—civilian version for PTSD, and Generalised Anxiety Disorder 2-item scale for anxiety. Two studies assessed PPE-related experiences among carers [30, 31]. The cohort of Nafilyan et al. [32] was based on Public Health Data Asset, whereas the cross-sectional analysis by Martin et al. [33] was conducted using data from the United Kingdom Research study into Ethnicity and COVID-19 Outcomes in carers (UK-REACH) cohort. Giebel et al. [23] and Hoernke et al. [30] performed thematic analyses of the impact of COVID-19 on care provision and the experiences of frontline healthcare workers, respectively.

## Discussion

The present scoping review identified 10 eligible studies and provided a broad overview of currently available evidence related to the impact of COVID-19 on ethnically minoritised carers. The results showed that most studies involved multiple healthcare occupations with a comparatively small representation from care homes. On the other hand, the studies focusing on participants from care homes had a small number of carers from the ethnically minoritised background. The evidence map revealed a significant gap in research exclusive for ethnically minoritised carers in UK care homes. This section discusses the experiences reported in included studies. The experiences of carers during COVID-19 were noted as related to mental disorders, inadequate availability of PPE, workplace discrimination, lack of trust in raising voices against concerns, future uncertainties, and problems in care delivery.

Multiple studies found anxiety, depression, stress, and PTSD among carers. In Melbourne et al.’s [27] study, participants from the ‘other’ (other than Asian, Black, mixed/multiple ethnicities) ethnic groups exhibited the highest odds of anxiety/depression compared to the White ethnic group. The odds ratio for anxiety/depression was less than 1 for Black, Asian, and mixed/multiple groups. However, these ethnic groups demonstrated higher odds for PTSD compared to participants in the white group. In terms of occupation type, the highest odds of PTSD were observed in nurses. Brewin et al. [26] surveyed to explore the best predictive symptoms of severe distress in UK health and social care staff, including nursing or carers. They found an increased rate of anxiety and depression and higher baseline stress levels than usual. In Greene et al.’s [28] study, approximately one-third of participants reported being moderate to highly stigmatised, and more than half were moderate to extremely worried about catching the COVID-19 infection. These worried carers demonstrated higher chances of meeting the criteria for PTSD. The majority of the carers working in UK hospitals, care or nursing homes, and other community settings met clinically significant levels of distress. Comparing PTSD between different work settings revealed higher levels of PTSD in nursing or carers. The indicators for worsening mental health among careers were associated with inadequate, inappropriate, and unequal PPE distributions [31]. They also had underlying anxiety about contracting the COVID-19 virus and perceptions of uncertainties related to care home futures. Shortage of staff resulted from isolation, aggravated stress, and burnout among carers. However, the sample size of ethnic minorities was insufficient to validate differences in mental disorders between carers from different ethnic groups.

Poor mental health was reported in association with workplace discrimination and inadequate PPE access. The odds of experiencing discrimination were two to three times higher in the ethnic minority than in the White. Blacks were more likely to work night shifts and had to work longer hours. Asians and Black had higher contact with COVID-19 patients but lower access to PPE [27]. Some studies noted hesitation among ethnically minoritised carers for raising voices against perceived concerns. For instance, in Melbourne et al.’s [27] study, carers lacked trust in their employers to address work-related concerns. Greene et al. [28] reported that a substantial proportion (30.2%) of 1194 total respondents from UK hospitals, nursing or care homes, and other community settings were unable to tell their team leader or manager if they were not coping with the COVID-19-related situation.

Studies reported a shortage of PPE throughout the UK, particularly in care homes, community health facilities, and general practice [30] and during the early days of the pandemic [29]. Besides inadequate supply, carers also lacked training on the safe use of PPE [30] and reported PPE as inappropriate for use. The most substantial risk of COVID-19 infection and clinically significant mental disorders were estimated among carers reporting limited, restricted, or unsuitable PPE and workplace discrimination [31, 34].

The experiences regarding PPE during COVID-19 in the UK highlighted difficulties in patient care with PPE. The tiring and uncomfortable wearing of PPE caused difficulty in breathing, rashes, bruises, dry skin, headaches and irritability. Although the carers persisted in care delivery, the laborious effects could become intense. In Hoernke et al.’s study, some healthcare workers expressed their desire to be reassigned to non-COVID-19 treatment wards because of the discomfort associated with wearing hot and sweaty full-length gowns [30]. The carers perceived that extreme cleaning procedures and the use of PPE often acted as barriers to their working roles [29]. The COVID-19-related restrictions for visitors to care homes challenged residents’ mental and physical health, posing extra workload to carers [23].

The risk of COVID-19 infection is not only higher among carers than in non-essential jobs, but carers also carry a risk of getting infection more substantial than in other essential jobs. Studies determining occupational differences for COVID-19 mortality in the UK showed higher risk among carers [32] and ethnically minoritised carers, particularly Black ethnicity [33], than other essential occupations and White ethnicity. The risk of infection and associated fear of containing the virus were also reported to be related to mental health and the availability of PPEs.

The carers in UK’s care homes faced uncertainties arising from postulated care home closures due to reporting increased COVID-19 cases and deaths in care homes. An infection outbreak in a care home could impact the decision-making of people considering long-term care, causing financial losses [23]. Moreover, the carers expressed fear about the future of care homes, postulating that increased work pressure and perceived lack of support would discourage people from working in this field [29].

Overall, the present scoping review identified that ethnically diverse carers experienced mental health challenges in terms of anxiety, depression, stress, and PTSD. The carers felt stigmatised due to fear of catching COVID-19, the higher risk of COVID-19 infection and mortality, and about uncertain future of care homes during the pandemic. In Greene et al.’s study, 77.6% of the UK frontline health and social care workers were moderately to extremely worried about passing the infection to others, and 36.5% expressed feeling moderately to extremely stigmatised [28]. Earlier studies also reported that healthcare workers concerned about being infected and passing the infection on to others might feel stigmatised because of their job roles [35–37]. The carers in Hanna et al.’s study raised concerns over the growing stigma of care homes after negative media reports regarding the high COVID-19-related casualties in care homes and the impact this will have on attracting residents to the care homes [29].

The available studies showed that ethnic minorities reported higher odds of experiencing discrimination at the workplace in terms of extra workload and limited supply of appropriate PPE, leading to a substantial risk of mental disorders. The carers faced challenges in care and comforting the residents due to protective measures and pandemic-related restrictions. Since the studies are limited for ethnically minoritised carers who make up a substantial proportion of the UK’s care home staff, the present scoping review identifies the need to further explore the COVID-19-related experiences exclusively of this subpopulation.

Since many studies largely included a sample of hospital settings carers, the results could only be related to the carer in care homes with an underrepresentation of ethnically minoritised staff. The job role of carers was not mentioned in the studies that included carers in nursing or care homes along with other healthcare occupations; this underpins the importance of capturing the views of a broader population of carers. Most of the interview-based studies were conducted online instead of face-to-face. Finally, the present scoping review lacked the included studies’ quality assessment, which might limit its scope.

## Conclusion

A scooping review was conducted to identify the evidence regarding the experiences of ethnically minoritised carers in UK care homes. The systematic search and identification resulted in 10 eligible studies. The studies have been conducted with diverse methodologies and various healthcare populations involving fewer carers from care homes and carers of ethnically minoritised backgrounds. The carers exhibited a high risk of COVID-19 infection, fear of containing the disease, limited PPE supply, lack of proper training for PPE use during the pandemic’s early phases, mental disorders, workplace discrimination, and uncertainties about their future as carers. More explorative and well-managed studies are warranted to increase the understanding of COVID-19-related experiences of ethnically minoritised carers in UK care homes.

## Data Availability

All data and material appear in article.
